# The Hypodermic Injection

**Published:** 1884-08

**Authors:** 


THE hypodermic injection of morphine has the same control over infantile convulsions that it exercises over eclampsia. Also it will generally prevent an epileptic paroxysm, whenever the near approach of one can be discovered.—*N. Y. Med. Journal.*

